# Indwelling catheter vs intermittent catheterization: is there a difference in UTI susceptibility?

**DOI:** 10.1186/s12879-023-08475-7

**Published:** 2023-08-02

**Authors:** Vera Neumeier, Fabian P. Stangl, Joëlle Borer, Collene E. Anderson, Veronika Birkhäuser, Oksana Chemych, Oliver Gross, Miriam Koschorke, Jonas Marschall, Shawna McCallin, Ulrich Mehnert, Helen Sadri, Lara Stächele, Thomas M. Kessler, Lorenz Leitner

**Affiliations:** 1 Department of Neuro-Urology, Balgrist University Hospital, University of Zürich, Forchstrasse 340, 8008 Zürich, Switzerland; 2 Department of Urology, Inselspital, University Hospital Bern, Bern, Switzerland; 3 Department of Urology, University Hospital Zürich, University of Zürich, Zürich, Switzerland; 4 Swiss Paraplegic Research, Nottwil, Switzerland; 5 Department of Health Sciences and Medicine, University of Lucerne, Lucerne, Switzerland; 6 Division of Infectious Diseases, Washington University School of Medicine, St. Louis Missouri, USA

**Keywords:** Asymptomatic bacteriuria, Intermittent catheterization, Indwelling catheter, Neurogenic lower urinary tract dysfunction, Urinary tract infection

## Abstract

**Background:**

Patients with neurogenic lower urinary tract dysfunction (NLUTD) often rely on some type of catheterization for bladder emptying. Intermittent catheterization (IC) is considered the gold standard and is preferred over continuous catheterization, since it is considered to cause fewer urinary tract infections (UTIs) than indwelling catheterization. The main objective of our study was to describe UTI prevalence (at visit) and incidence (within the last 12 months) and urine culture characteristics between patients using an indwelling catheter versus (*vs*) those performing IC.

**Methods:**

In this cross-sectional study, we prospectively evaluated from 02/2020 to 01/2021 patients with NLUTD undergoing urine cultures for prophylactic reasons or due to UTI symptoms. At visit, all patients underwent a standardized interview on current UTI symptoms as well as UTI history and antibiotic consumption within the past year. Patients using an indwelling catheter (*n* = 206) or IC (*n* = 299) were included in the analysis. The main outcome was between-group differences regarding UTI characteristics.

**Results:**

Patients using an indwelling catheter were older (indwelling catheter *vs* IC: median 66 (Q1-Q3: 55—77) *vs* 55 (42—67) years of age) and showed a higher Charlson comorbidity index (indwelling catheter *vs* IC: median 4 (Q1-Q3: 2–6) *vs* 2 (1–4) (both *p* < 0·001).

A total of 40 patients from both groups were diagnosed with a UTI at visit (indwelling catheters *vs* IC: 8% (16/206) *vs* 8% (24/299); *p* = 0·782), and the number of UTIs within the past 12 months was not significantly different between groups. Overall, *Escherichia coli* (21%), *Enterococcus faecalis* (17%), and *Klebsiella* spp. (12%) were the most frequently detected bacteria.

**Conclusions:**

In this cohort of patients with NLUTD, we did not find relevant differences in UTI frequency between groups. These results suggest that UTI-related concerns should not be given undue emphasis when counseling patients for catheter-related bladder emptying methods.

**Supplementary Information:**

The online version contains supplementary material available at 10.1186/s12879-023-08475-7.

## Background

Many patients with neurological disease present with bladder storage and voiding symptoms, a situation summarized under the term neurogenic lower urinary tract dysfunction (NLUTD) [1]. Optimal long-term management of the lower urinary tract often requires assisted bladder emptying and the use of some type of catheterization [1, 2]. Major guidelines recommend intermittent catheterization (IC) over continuous catheterization if cognition and manual dexterity allow for it [2, 3]. Besides an improved quality of life and sexual well-being, IC is thought to cause fewer urethral complications, less stone disease, less upper urinary tract damage, and does not require health care visits for catheter changes, compared to indwelling catheters [4]. In addition to these advantages, IC is thought to cause fewer urinary tract infections (UTIs) than indwelling catheterization, an argument often prioritized in patient counseling [2, 3]. However, only limited data is available to support this assumption.

Recurrent UTIs are a major problem in patients with NLUTD, as they negatively influence health-related quality of life and have potential life-threatening consequences [2, 5]. Diagnostic challenges include an overlap of symptoms typically regarded as UTI-related, such as urinary urgency, frequency, and loss of urine. Moreover, individuals with a spinal cord injury (SCI) might not report pain and dysuria at all, making UTI diagnosis more difficult [2]. The requirement of an indwelling catheter or IC additionally complicates the situation further for clinical decision-making, as bacteriuria, leukocyturia, hematuria, and positive nitrite are common findings [6]. While there is an agreement that asymptomatic bacteriuria (ABU) in catheterized patients should not be treated [2, 3, 5], no consistent definition of UTI in patients with NLUTD exists and no general recommendations for when antibiotics should be used to treat ambiguous symptoms are available [6–8]. This often results in overtreatment with antibiotics and significant strain on the patient, the caregiver, and the healthcare system, not only economically [9], but also increasing the risk for the development of multidrug-resistant organisms [10].

The goal of this study was to describe differences in UTI frequency and microbiological characteristics between patients using continuous catheterization and those performing IC.

## Methods

### Patients

**Fig. 1:**
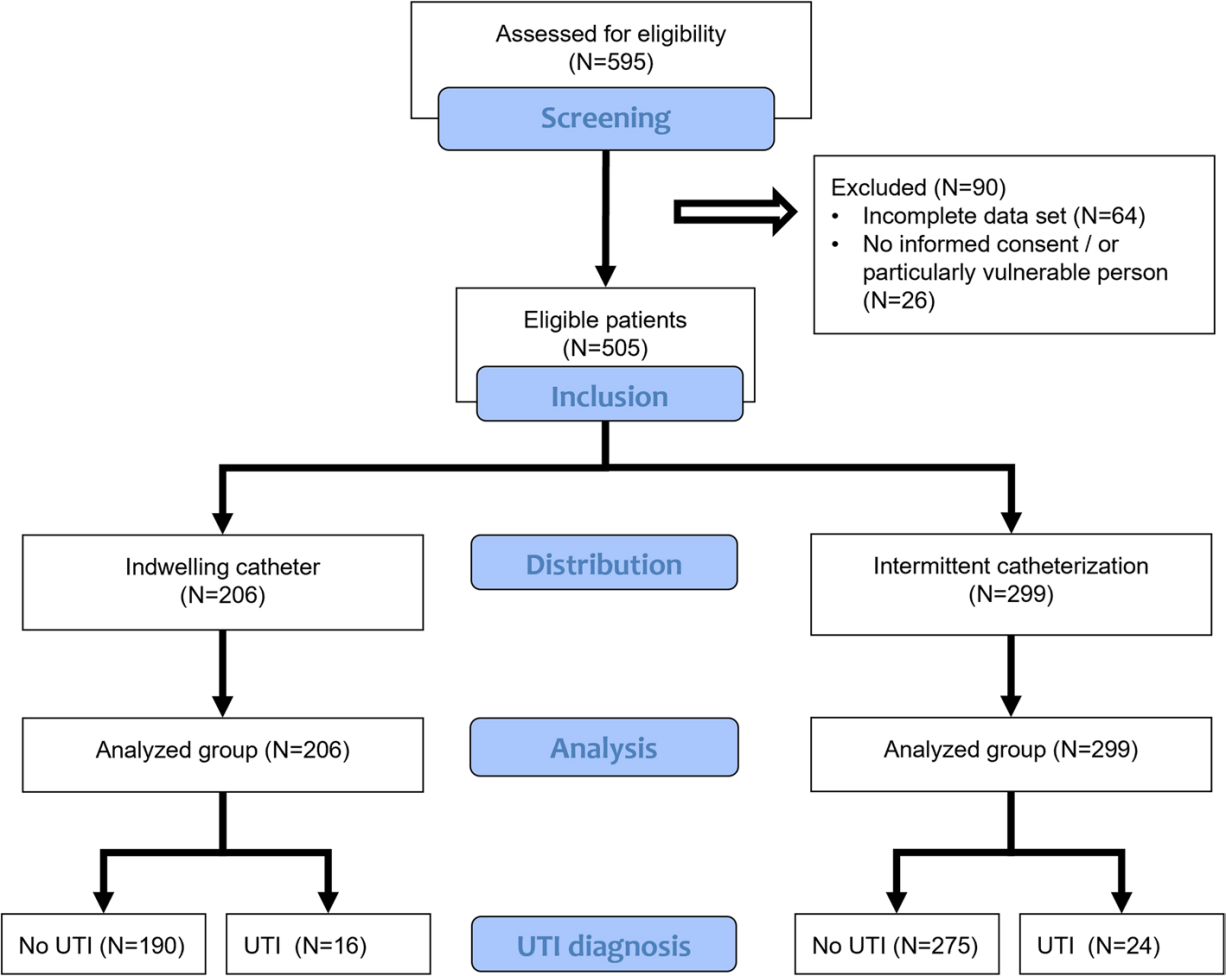
Patient flow chart. Flow chart of included patients between 02/2022 and 01/2021 divided for bladder management and presence of a urinary tract infection (UTI)

Between 02/2020 and 01/2021, a consecutive series of 595 patients with NLUTD relying on any type of catheterization and undergoing urine culture for any reason were evaluated in a cross-sectional fashion at our tertiary neuro-urology department (Balgrist University Hospital, University of Zürich, Zürich, Switzerland) for participation in this study (Fig. 1). Of these, 505 patients (37% (188/505) females and 63% (317/505) males) could be included. In our department, patients with an indwelling catheter are instructed to regularly clamp the catheter during the day to preserve bladder capacity (if possible) and to leave the catheter on continuous drainage during the night for comfort reasons. Indwelling catheters are generally changed every 6–8 weeks. Patients relying on intermittent catheterization are trained to perform catheterization 4–6 times in 24 h. In both groups, recommended urinary bladder target volumes are between 350 and 550 mL. Study exclusion criteria were age < 18 years, incomplete data sets (e.g., incomplete medical history or laboratory/microbiological results), and particularly vulnerable persons. This study was approved by the Cantonal Ethics Committee Zürich, Switzerland (BASEC-Nr. 2021–02401). All patients provided written informed consent, for the reuse of medical data for research purposes. This study was performed in accordance with the World Medical Association Declaration of Helsinki and in line with the International Conference on Harmonisation (ICH), Good Clinical Practice (GCP) Guidelines (E6) and the International Organization for Standardization (ISO, 14,155:2021–5). STROBE (Strengthening of Reporting of Observational Studies in Epidemiology; see Appendix) guidelines were used to promote quality reporting.

### Investigation and intervention

All subjects underwent neuro-urological assessment, including patient history and clinical examination [1]. If urine culture was indicated (i.e., for prophylactic reasons prior to invasive diagnostics and/or surgery or due to UTI symptoms), patients were asked to provide a written informed consent and underwent a structured interview on UTI history based on the International Spinal Cord Injury Urinary Tract Infection Basic Data Set including additional questions on UTI frequency and antibiotic consumption within the last 12 months [11].

Patients with an indwelling catheter had the catheter replaced prior to urine culturing, and in all other patients, urine was collected by sterile in- and out-catheterization. Urine cultures were analyzed in compliance with international recommendations (ISO/IEC 17025:2017 and SN EN ISO/IEC 17025:2018), and bacteriuria was defined by the presence of ≥ 10^3^ colony forming units (CFU) in the urine [12]. Pathogen identification on urine cultures was performed by microscopy, standard biochemical identification methods, and matrix-assisted laser desorption time-of-flight mass spectrometry (MALDI-TOF). Antimicrobial resistance was identified in compliance with Clinical and Laboratory Standards Institute (CLSI) Guidelines and classified following the European Committee on Antimicrobial Susceptibility Testing (EUCAST) Guidelines [13].

The presence of UTI at visit was defined as a positive urine culture with ≥ 10^3^ CFU in combination with at least one clinical symptom suggestive of UTI, including fever, hematuria, and bladder or flank pain without any other discernible cause, as well as the new onset or acute worsening of urgency, incontinence, increased catheterization frequency, unexplained spasticity, or autonomic dysreflexia. Solely the presence of pyuria, cloudy, or foul-smelling urine was not regarded as a UTI, but was still recorded and these cases were classified as ABU. ABU was defined as the presence of ≥ 10^3^ CFU in urine specimens of patients without any of the symptoms/signs described above referable to UTI [14].

All subjects were interviewed on how many antibiotic therapies were prescribed (by primary care providers and/or specialists) within the last 12 months for (suspected) UTIs or for any other indications. To evaluate UTI incidence, any antibiotic therapy prescribed for (suspected) UTIs within the last 12 months was counted as UTI, irregardless of availability of a positive urine culture [2, 5].

### Outcomes

The primary outcome was the prevalence of UTI at the time of patient visit for patients using an indwelling catheter in comparison to patients performing IC.

The secondary outcomes consisted of number of UTI diagnoses/number of antibiotic therapy cycles for UTI during the previous 12 months, uropathogen characteristics, and antibiotic susceptibility patterns within and between groups, as well as clinical and microbiological factors associated with UTI. To account for potential recall bias, only the number of UTIs within the last 12 months is reported instead of using the definition of recurrent UTIs (i.e. ≥ 2 infections in 6 months or ≥ 3 infections in one year).

### Statistical analysis and data management

Data distribution was tested by Q-Q plots. Normally distributed data are presented as mean ± standard deviation (SD), non-normal data as median and 25^th^ (Q1) and 75^th^ (Q3) percentile, and categorical data as numbers and percentages. For comparisons between unrelated samples (i.e., patients using an indwelling catheter *vs* patients performing IC), the unpaired t-test was used for approximately normally distributed data and the non-parametric Mann–Whitney U test for non-normal distributed data. The chi-square-test or the Fisher's exact test was used for comparison of unrelated binary data where applicable.

Univariate logistic regressions were performed to analyze the association between type of catheterization, sex, Charlson comorbidity index (CCI), number of UTIs in the past 12 months, and age. Odds ratios (OR) are presented with corresponding 95% confidence intervals (CI) for the coefficient estimates. Results are presented both unadjusted and adjusted for the method of bladder emptying. A *p*-value of < 0·05 was considered statistically significant. Analyses were conducted in R version 4.1.2 (2021–11-01) (R: A language and environment for statistical computing. R Foundation for Statistical Computing, Vienna, Austria. https://www.R-project.org/.)

### Findings

Patient characteristics are presented in Table 1. The indwelling catheter group consists of both, 34% (70/206) of patients with a transurethral catheter and 66% (136/206) of patients with a suprapubic catheter. Overall, no meaningful clinical or microbiological differences were found between the patients with a transurethral versus suprapubic catheter, so they were considered as a single group, i.e. patients with an indwelling catheter and compared to those performing IC. Detailed information on patients with an indwelling (transurethral and suprapubic) catheter are provided in the supplement (including Supplement Table 1, Supplement Table 2, Supplement Table 3, and Supplement Fig. 1).

**Table 1:** Characteristics of the study population

		Method of bladder emptying	
	Total	Indwelling catheter	Intermittent catheterization
	(*N* = 505)	(*N* = 206)	(*N* = 299)
**Age, years, median (Q1-Q3)**	59 (47–72)	66 (55–77)	55 (42–67)
**Sex, n (%)**			
Female	188 (37%)	75 (36%)	113 (38%)
Male	317 (63%)	131 (64%)	186 (62%)
**Cause of NLUTD, n (%)**^**a**^			
Spinal cord injury	230 (45%)	105 (51%)	125 (42%)
Tetraplegia	77 (15%)	51 (25%)	26 (9%)
Paraplegia	153 (30%)	54 (26%)	99 (33%)
Spinal canal stenosis	76 (15%)	36 (18%)	40 (13%)
Multiple sclerosis	40 (8%)	16 (8%)	24 (8%)
Conus cauda syndrome	31 (6%)	3 (2%)	28 (9%)
Stroke	27 (5%)	19 (9%)	8 (3%)
Parkinson's disease	21 (4%)	17 (8%)	4 (1%)
Spina bifida	19 (4%)	2 (1%)	17 (6%)
Polyneuropathy	19 (4%)	10 (5%)	9 (3%)
Other neurological disorders	123 (25%)	39 (19%)	84 (28%)
**CCI, median (Q1-Q3)**	3 (2–5)	4 (2–6)	2 (1- 4)
**Locomotion, n (%)**			
Walking	263 (52%)	62 (30%)	201 (67%)
Wheelchair user	230 (46%)	133 (65%)	97 (32%)
Bed ridden	12 (2%)	11 (5%)	1 (0%)
**Urine culture, n (%)**			
Bacterial growth	428 (85%)	189 (93%)	239 (80%)
UTI	40 (8%)	16 (8%)	24 (8%)
Asymptomatic bacteriuria	388 (77%)	173 (84%)	215 (72%)
No bacterial growth	77 (15%)	17 (8%)	60 (20%)
**UTI prophylaxis, n (%)**			
Bladder irrigation	96 (19%)	39 (19%)	57 (19%)
Oral antibiotics	5 (1%)	0 (0%)	5 (2%)
Non antibiotic oral prophylaxis^b^	41 (8%)	10 (5%)	31 (10%)

*CCI* Charlson comorbidity index, *NLUTD* Neurogenic lower urinary tract dysfunction, *UTI* Urinary tract infections

^a^One patient can present with more than one neurological diagnosis causing NLUTD. Other neurological disorders consisted of amyotrophic lateral sclerosis, Arnold Chiari malformation, Brown-Séquard syndrome, brain tumor, cerebral palsy, encephalitis, epilepsia, Guillain-Barré syndrome, hydrocephalus, Morbus Friedreich, multi system atrophy, peripheral nerve lesion, tethered cord syndrome, traumatic brain injury, and others

^b^Urine acidifiers, herbal extracts, D-mannose, and others

**Table 2:** Association between clinical parameters and UTI diagnosis. Logistic regression of UTI diagnosis at the time of patient visit with and without adjustment for catheter type

	**unadjusted OR (95% CI)**	*p*-value	**adjusted OR (95% CI)**	*p*-value
**Method of bladder emptying**		0.92		
Indwelling catheter	0.96 (0.49–1.85)		not applicable	
IC	reference			
**Sex**		0.97		0.99
Female	reference		reference	
Male	0.99 (0.51–1.97)		0.99 (0.51–1.97)	
**CCI**		0.29		0.58
≥ 3	1.24 (0.41–5.42)		1.24 (0.39–5.49)	
2	2.51 (0.76–11.39)		2.51 (0.75–11.40)	
1	2.16 (0.54–10.67)		2.15 (0.53–10.71)	
0	reference		reference	
**UTI frequency (in the past 12 months)**		< 0.01		0.02
≥ 3	15.11 (5.89–42.25)		15.10 (5.88–42.25)	
2	6.34 (2.18–18.87)		6.35 (2.18–18.93)	
1	3.96 (1.52–11.01)		3.96 (1.52–11.01)	
0	reference		reference	
**Age**	0.99 (0.970–1.010)	0.44	0.99 (0.97–1.01)	0.74

*CCI* Charlson comorbidity index, *CI* Confidence interval, *IC* Intermittent catheterization, *OR* Odds ratio, *UTI* Urinary tract infection

**Table 3:** Distribution of isolated bacteria in urine cultures and antibiotic resistances

a) Bacterial distribution		Indwelling catheter		Intermittent catheterization	
	Total (*N* = 868) n (%)	UTI (*N* = 37) n (%)	ABU (*N* = 472) n (%)	UTI (*N* = 29) n (%)	ABU (*N* = 330) n (%)
*Escherichia coli*	181 (21%)	3 (8%)	61 (13%)	17 (59%)	100 (30%)
*Enterococcus faecalis*	147 (17%)	11 (30%)	102 (22%)	2 (7%)	32 (10%)
*Klebsiella* spp.	106 (12%)	4 (11%)	43 (9%)	3 (10%)	56 (17%)
*Streptococcus viridans*	59 (7%)	3 (8%)	15 (3%)	2 (7%)	39 (12%)
*Pseudomonas aeruginosa*	40 (5%)	3 (8%)	32 (7%)	0 (0%)	5 (2%)
*Aerococcus urinae*	40 (5%)	0 (0%)	29 (6%)	0 (0%)	11 (3%)
*Streptococcus anginosus*	28 (3%)	0 (0%)	9 (2%)	0 (0%)	19 (6%)
*Staphylococcus aureus*	26 (3%)	0 (0%)	22 (5%)	0 (0%)	4 (1%)
*Staphylococcus epidermidis*	23 (3%)	1 (3%)	11 (2%)	0 (0%)	11 (3%)
*Proteus mirabilis*	22 (3%)	1 (3%)	20 (4%)	0 (0%)	1 (0%)
others	196 (23%)	11 (30%)	128 (27%)	5 (17%)	52 (16%)
**b)** **Detected** **antibiotic** **resistances**		*Escherichia coli* (*N* = 181)		*Klebsiella* spp. (*N* = 105)	*Enterococcus faecalis* (*N* = 147)
Total of tested bacterial species					
Amoxicillin / Clavulanic acid		30% (55/181)		18% (19/105)	n.a
Quinolones		5% (9/181)		9% (9/105)	6% (9/147)
Cotrimoxazole		38% (69/181)		16% (17/105)	n.a
Nitrofurantoin		1% (2/181)		n.a	n.a
3rd gen. Cephalosporin		9% (16/181)		6% (6/105)	n.a
Carbapenems		0% (0/181)		0% (0/105)	n.a

*ABU* Asymptomatic bacteriuria, *UTI* Urinary tract infection

The most common underlying diagnoses for NLUTD were spinal cord injury (SCI), spinal canal stenosis, and multiple sclerosis. Patients using an indwelling catheter were older (median: 66 (Q1-Q3: 55—77) *vs* 55 (42—67) years of age; *p* < 0·001), and had a higher CCI (median: 4 (Q1-Q3: 2—6) *vs* 2 (1—4); *p* < 0·001) than patients performing IC. In case of SCI, patients using an indwelling catheter presented with a higher lesion level (indwelling catheter *vs* IC: tetraplegia 25% (51/206) *vs* 9% (26/299); paraplegia 26% (54/206) *vs* 33% (99/299); *p* < 0.001).

At the time of patient visit, a UTI was diagnosed in 8% (16/206) of patients using an indwelling catheter and 8% (24/299) of patients performing IC (*p* = 0·782). In case of UTI, reported symptoms included bladder pain (70%, 28/40), new onset or acute worsening of urgency (43%, 17/40), cloudy urine (43%, 17/40), foul smelling urine (35%, 14/40), fever (25%, 10/40), new onset of incontinence (20%, 8/40), unexplained spasticity (13%, 5/40), lethargy and feeling of unease (13%, 5/40), hematuria (10%, 4/40), kidney pain (8%, 3/40), nausea and vomiting (3%, 3/40), and others (13%, 5/40).

At least one UTI was diagnosed in 46% (95/206) and 44% (131/299) (*p* = 0·674) of patients > 2 UTIs within the last 12 months were found in 8% (17/206) and 11% (33/299) (*p* = 0·380) of patients using an indwelling catheter or performing IC, respectively. Frequently prescribed antibiotics for past UTIs were beta-lactams (penicillins or cephalosporins) (11%, 57/505), quinolones (7%, 33/505), and sulfonamide/trimethoprim (4%, 20/505). A UTI within the past 12 months was the only factor that was significantly associated (*p* < 0·001) with UTI diagnosis at visit (Table 2).

**Fig. 2:**
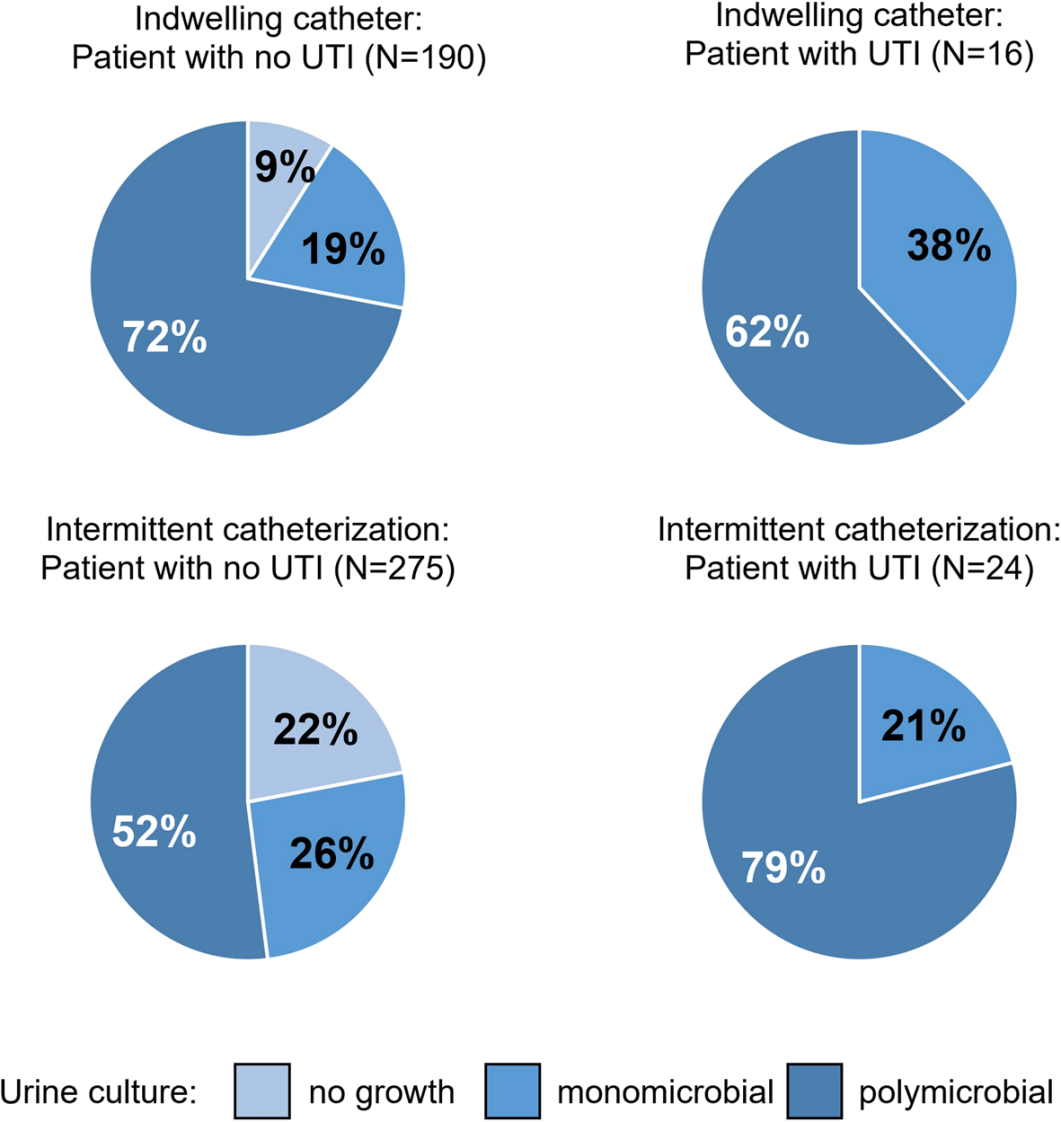
Urine culture characteristics. Urine culture results show percentages of patients without bacterial growth (no growth), detection of one species of bacteria (monomicrobial), or multiple bacterial species (polymicrobial) in patients using an indwelling catheter or performing intermittent catheterization

Microbial growth was found in 85% (428/505) of the urine cultures, of which 8% (40/505) were according to our definition UTIs and 77% (388/505) were ABU. Polymicrobial growth was found in 52% (224/428) of all positive cultures and was significantly more prevalent in patients using an indwelling catheter than IC (indwelling catheter *vs* IC 78% (147/189) *vs* 32% (77/239); *p* < 0·001) (Fig. 2).

From the 428 positive urine cultures 868 pathogens were isolated. Overall, *Escherichia coli* (21% (171/868)), *Enterococcus faecalis* (17% (147/868)), and *Klebsiella* spp. (12% (106/868)) were the most commonly detected bacterial species. *E. faecalis* was more frequently found in patients using an indwelling catheter, while *E. coli* was more frequently found in patients performing IC (*p* = 0·011) (Table 3). Resistance rates for the commonly prescribed antibiotics were moderate, ranging from 1%-38% (Table 3).

## Discussion and interpretation

### Main findings

When investigating a cohort of over 500 patients with NLUTD relying on any type of catheterization for bladder emptying, no differences in UTI prevalence (at visit) or incidence (within the last 12 months) were found between patients using an indwelling catheter or performing IC. While IC is the preferred management for patients with NLUTD who cannot effectively empty their bladders because it is associated with fewer general complications [15], there seems to be no reason to choose one catheterization approach over the other from an infection risk perspective. However, significant between-group differences in bacterial species distributions and the presence of mono- versus polymicrobial urine cultures were detected. These findings indicate that the type of bladder catheterization might influence microbial population characteristics. Importantly, as detected bacterial species vary significantly between patients with NLUTD, and even more from the non-neurological population [5], urine culture is mandatory in any UTI treatment protocol for catheterizing patients.

### Findings in context of existing evidence

UTIs are a major problem in patients with NLUTD, and incidence ranges from 1–10/year depending on the definition of UTI [16]. In the SCI population, UTI remains a significant health burdens and accounts, according to a retrospective cohort study, for more than 50% of all emergency visits [17] and most infection-related hospitalizations [18]. In our cohort, less than 10% reported > 2 infections within the last 12 months indicating a comparatively low incidence compared to available literature. However, the overall ABU rate (77%, 388/505) was comparable with existing evidence [2, 14]. There are various reasons for this: foremost, there is no internationally accepted standardized definition for UTIs in patients with NLUTD or for patients performing IC [8], and relevant heterogeneity for both clinical and laboratory criteria exists [8], which hinders comparison. Berger et al. [20] demonstrated that depending on the definition the rate of diagnosed UTIs in the same cohort is 14–45%. Our conservative approach and prudent use of antibiotic therapy, in line with a strict antibiotic stewardship program in our department, might have further contributed to a restrictive UTI diagnosis. The influence of UTI-prophylactic measures used by 28% of our patients on these results remain uncertain, especially as prophylaxis was more common in patients with recurrent infections.

Indwelling catheterization is generally considered to increase the risk for UTI compared to IC; as supported by a systematic review including 2321 patients [21]. However, in our cohort of over 500 patients, no significant differences were observed between the methods of catheter-based bladder drainage. Upon closer evaluating of the systematic review [21], only two of six included studies comparing indwelling transurethral catheterization with IC and only one of four studies comparing indwelling suprapubic catheterization with IC revealed significant differences. Notably, one of the studies contributing significant results, which had a major impact on promoting IC [22], was only available as conference abstract at the time of literature search. Insights from the later published full article revealed that data on UTI frequency was assessed retrospectively in a highly subjective manner, not by providing patients a clear definition of UTI but by asking “How many UTIs (including bladder or kidney infections) have you had over the past 12 months?” [22], in our cohort antibiotic consumption was a prerequisite for past UTIs. Furthermore, one retrospective study presenting a significant difference between emptying methods, published by McGuire and Savastano in 1986 [23], reported febrile UTI in 92% (12/13) and 32% (7/22) of female SCI patients using an indwelling catheter or performing IC, respectively, within a mean follow-up period of seven years. While all patients with an indwelling catheter presented with bladder stone complications, none of the patients in the IC group presented with bladder stones. In our study, only 2% (10/505) of patients suffered from UTI-related febrile complications, indicating relevant differences in patient selection compared to McGuire’s work.

The most commonly encountered bacteria in our study were *E. coli*, *E. faecalis*, and *Klebsiella* spp. In contrast, our previous study [6] detected *Proteus mirabilis* more often than *E. faecalis*; however, this study additionally included spontaneous voiders (35%) and the fraction of patients relying on indwelling catheters (21%), which harbored significantly more *E. faecalis*, was low. A chart review by Pannek et al. [24] in NLUTD patients showed similar results for the five most common detected bacteria in the outpatient setting, and comparable bacterial distribution and abundance. The general bacterial spectrum and resistance rates of our cohort depicts comparable results in the context of existing literature investigating antibiotic resistance in central Europe [24]. Since bacterial resistance is distinctly dependent on regional factors influencing resistance rates, broadly implemented antibiotic stewardship positively impacts antibiotic resistance [25]. Compared to patients suffering from an acute uncomplicated cystitis, where *E. coli* is the most frequent uropathogen (75–95%) and other bacteria (mainly *Enterobacteriaceae*) are detected only occasionally [26], the variation in the NLUTD population is different.

### Implications for practice

The majority of our patients exhibited bacteriuria. According to all major guidelines [2, 3, 5, 14] ABU generally does not require treatment, and the diagnosis of a UTI should consider both clinical symptoms and laboratory findings. However, evidence shows that a positive urine culture remains a significant predictor for antibiotic prescription after routine urine testing in the SCI population [27]. Therefore, urine cultures should be performed with clear indications. Given the rising threat of multidrug-resistant bacteria, appropriate use of antibiotics (i.e., antibiotic stewardship) is crucial, especially for vulnerable populations such as patients with NLUTD. The impact of our strict antibiotic stewardship program on resistance rates (0%-38%) for commonly prescribed antibiotics, cannot be answered with our study design. Nevertheless, to prevent antibiotic resistance systemic treatment of pyuria, cloudy, and malodorous urine should be avoided. Symptoms suggestive for UTI and reported in 3%-70% of our patients include fever, new or increased in incontinence, increased spasticity, malaise, lethargy or sense of unease, discomfort or pain over the kidney or bladder, or autonomic dysreflexia [2].

In patients with NLUTD urine culture with antibiotic sensitivity testing is essential for clinically suspected UTI, as the bacterial spectrum and antibiotic resistance may differ from the general population. Empirical antibiotic treatment should be considered for severe symptoms, while non-antibiotic treatment may be an appropriate first-line in afebrile patients. However, it should be noted that UTIs rarely can present with negative culture. In case of otherwise unexplained persistent symptoms short term clinical follow-up and repetitive cultures might be needed, given the potential atypical presentation in patients with NLUTD. Prophylactic measures should be implemented to avoid recurrence, including education of healthcare professionals and patients about distinction between ABU and UTI. Optimal lower urinary tract management, treatment of neurogenic detrusor overactivity, proper catheter use, and appropriate catheter material selection are crucial in minimizing misdiagnosis of UTI [19].

### Implications for research

The results prompt further inquiry into non-antibiotic options for acute UTI and prophylaxis in patients with NLUTD. Bladder irrigations or installations have repetitively shown potential in UTI prevention [28], however, well-structured trials confirming long-term efficacy and safety are missing. Available evidence for use of probiotics, phytotherapy, D-mannose, or immunostimulation (bacterial lysate of *E. coli*, e.g., UroVaxom®, OM Pharma SA, Geneva, Switzerland, or whole cell-inactivated bacteria, e.g., Uromune™, Inmunotek, Madrid, Spain) in patients with catheter-dependent bladder management is scarce.

Renewed interest in the use of bacteriophages, an accepted therapy in several Eastern European countries, could be a promising future treatment option [29, 30]. To further reduce antibiotic consumption, the necessity of antibiotic prophylaxis for invasive diagnostics and minimal invasive treatments in presence of ABU should be explored [31].

Despite higher incidence of polymicrobial urine cultures in patients with indwelling catheters no difference in UTI frequency could be found. This raises the question about factors other than bacterial colonization and potential existence of a protective eubiotic bacterial colonization.

In our department, patients with an indwelling catheter are instructed to regularly clamp the catheter during the day. While clamping of indwelling catheters should be avoided for short-term catheterization (< 7 days) as it can increase urinary complications [32] benefits for long-term management are unclear. Although clamping may generally be preferred by patients [33], additional research is required to further investigate its effects and optimize catheter management strategies.

### Limitations

This cross-sectional study was conducted at a highly specialized tertiary academic center with rigorous antibiotic stewardship programs. The involvement of highly trained neuro-urologists and health professionals may limit generalizability to other settings. To avoid potential selection bias, we aimed to include all patients applicable for the study, however, 15% of patients could not be consented, mostly for medical reasons or incomplete datasets (e.g., missing urine culture). Additional visits for UTI related problems in a primary care facility can further not be ruled out. Recall bias may have affected the reported frequency of UTIs within the past 12 months, however, to avoid underestimation of incidence a less rigorous definition was used.

The patients included in this study represent a highly heterogeneous population with vastly different treatment regimens that may lead to a possible inherent skew regarding resistance patterns and rate of UTI episodes. Over 50% of our patients have a SCI as the underlying cause of neurogenic NLUTD.

An a priori power calculation was not performed and we acknowledge that a difference in UTI prevalence between the indwelling catheter and IC users is not excluded by the 95%-CI produced by our estimates. However, we collected data from a large sample, and we felt the similarity of outcomes was a good representation of the clinical reality.

## Conclusions

In our patient population with NLUTD, we did not find relevant differences in UTI frequency for patients using an indwelling catheter compared to those performing IC, however, characteristics of microbiological findings were different between groups. While IC is the preferred management for patients with NLUTD who cannot effectively empty their bladders, as it is associated with fewer general complications, infectious outcomes seem to be similar with patients using an indwelling catheter. The microbiological characteristic further indicate that urine cultures are mandatory in case of UTIs prior antibiotic therapy in this population.

## Supplementary Information

**Additional file 1.**

**Additional file 2.**

## Data Availability

Individual participant data that underlie the results reported in this article, after de-identification will be available to researchers who provide a methodologically sound proposal from the publication date until 5 years after article publication, to achieve aims in the approved proposal. Proposals should be directed to the corresponding author (LL) and the requested data will be sent if applicable.

## Abbreviations

ABU: Asymptomatic bacteriuria
CCI: Charlson comorbidity index
CFU: Colony forming units
CI: Confidence intervals
CLSI: Clinical and Laboratory Standards Institute
EUCAST: European Committee on Antimicrobial Susceptibility Testing
GCP: Good Clinical Practice
IC: Intermittent catheterization
ICH: International Conference on Harmonisation
MALDI-TOF: Matrix-assisted laser desorption time-of-flight mass spectrometry
NLUTD: Neurogenic lower urinary tract dysfunction
SCI: Spinal cord injury
SD: Standard deviation
UTI: Urinary tract infection
