# Supplementary material for: Indwelling catheter vs intermittent catheterization: is there a difference in UTI susceptibility?
Source: BMC Infect Dis. 2023 Aug 2;23:507. doi: 10.1186/s12879-023-08475-7 (PMC10398982; doi:10.1186/s12879-023-08475-7)
Supplement: Supplementary file 2 — Additional file 2. [file 12879_2023_8475_MOESM2_ESM.docx]

**Supplement:** **Indwelling catheter vs intermittent catheterization: Is there a difference in UTI susceptibility?**

**Comparison of patients with an indwelling catheter**

In this study, we compared the prevalence, incidence, and characteristics of urinary tract infections (UTIs) in patients with neurogenic lower urinary tract dysfunction (NLUTD) who used either an indwelling catheter or performed intermittent catheterization. The indwelling catheter group included patients with a transurethral catheter (n=70) and a suprapubic catheter (n=136). We also compared these two subgroups in this supplement.

Patient characteristics are presented in Supplement Table 1. At the time of the patient visit, 9% (6/70) of patients using a transurethral catheter and 7% (10/136) of patients with a suprapubic catheter had a UTI (p=0·787). The number of UTIs within the past 12 months appeared to be associated with the odds of having a UTI at the visit, regardless of the type of indwelling catheter (Supplement Table 2). At least one UTI was diagnosed in 61% (43/70) and 38% (52/136) (p=0·0028) of patients using a transurethral catheter or a suprapubic catheter, respectively. More than two UTIs within the last 12 months were diagnosed in 11% (8/70) and 7% (9/136) (p=0·236) of patients using a transurethral catheter or a suprapubic catheter, respectively.

The bacterial distribution of patients with an indwelling catheter is presented in Supplement Table 3. Both patients with an indwelling catheter and with a suprapubic catheter typically had polymicrobial bacterial growth in urine cultures (Supplement Figure 1).

Remarkably, while the prevalence of UTIs was similar between subjects with transurethral and suprapubic catheters, the incidence of at least one UTI within the past 12 months differed significantly between the two groups. However, UTI frequency was the only risk factor associated with the presence of a UTI at the visit in both cases, unadjusted and adjusted for the type of indwelling catheter. Overall, the clinical and microbiological findings for both groups appeared to have no meaningful clinical differences, which in our opinion justify combining both groups into one (i.e. indwelling catheter) for comparison with intermittent catheterization, as done in the main manuscript.

**Supplement Table 1**: Characteristics of the study population relying on an indwelling catheter.

|  |  | Type of indwelling catheter | |
| --- | --- | --- | --- |
|  | Total | Transurethral catheter | Suprapubic catheter |
|  | (N=206) | (N=70) | (N=136) |
| **Age, years, median (Q1-Q3)** | 66 (55-77) | 71 (59-77) | 63 (52-76) |
| **Sex, n (%)** | | | |
| Female | 75 (36%) | 26 (37%) | 49 (36%) |
| Male | 131 (64%) | 44 (63%) | 87 (64%) |
| **Cause of NLUTD, n (%)*** | | | |
| Spinal cord injury | 105 (51%) | 34 (49%) | 71 (52%) |
| Tetraplegia | 51 (25%) | 16 (23%) | 35 (38%) |
| Paraplegia | 54 (26%) | 18 (26%) | 36 (27%) |
| Spinal canal stenosis | 36 (18%) | 18 (26%) | 18 (13%) |
| Multiple sclerosis | 16 (8%) | 2 (3%) | 14 (10%) |
| Conus cauda syndrome | 3 (2%) | 0 | 3 (2%) |
| Stroke | 19 (9%) | 9 (13%) | 10 (7%) |
| Parkinson's disease | 17 (8%) | 5 (7%) | 12 (9%) |
| Spina bifida | 2 (1%) | 2 (3%) | 0 |
| Polyneuropathy | 10 (5%) | 6 (9%) | 4 (3%) |
| Other neurological disorders | 39 (19%) | 17 (24%) | 22 (16%) |
| **CCI, median (Q1-Q3)** | 4 (2-6) | 5 (3-6) | 3 (2-6) |
| **Locomotion, n (%)** | | | |
| Walking | 62 (30%) | 31 (44%) | 31 (23%) |
| Wheelchair user | 133 (65%) | 31 (44%) | 102 (75%) |
| Bed ridden | 11 (5%) | 8 (11%) | 3 (3%) |
| **Urine culture, n (%)** | | | |
| Bacterial growth | 189 (93%) | 58 (83%) | 131 (96%) |
| UTI | 16 (8%) | 6 (9%) | 10 (7%) |
| Asymptomatic bacteriuria | 173 (84%) | 52 (74%) | 121 (89%) |
| No bacterial growth | 17 (8%) | 12 (17%) | 5 (4%) |
| **UTI prophylaxis, n (%)** | | | |
| Bladder irrigation | 39 (19 %) | 7 (10%) | 32 (24%) |
| Oral antibiotics | 0 (0%) | 0 | 0 |
| Non antibiotic oral prophylaxis** | 10 (5%) | 3 (4%) | 7 (5%) |
| [CCI=Charlson comorbidity index, NLUTD=neurogenic lower urinary tract dysfunction,](https://www.mdcalc.com/calc/3917/charlson-comorbidity-index-cci) UTI=urinary tract infections. *One patient can present with more than one neurological diagnosis causing NLUTD. Other neurological disorders consisted of amyotrophic lateral sclerosis, Arnold Chiari malformation, Brown-Séquard syndrome, brain tumor, cerebral palsy, encephalitis, epilepsia, Guillain-Barré syndrome, hydrocephalus, Morbus Friedreich, multi system atrophy, peripheral nerve lesion, tethered cord syndrome, traumatic brain injury, and others. **Urine acidifiers, herbal extracts, D-mannose, and others. | | | |

**Supplement Table 2**: Association between clinical parameters and UTI diagnosis. Logistic regression of UTI diagnosis at the time of patient visit with and without adjustment for catheter type (transurethral catheter vs. suprapubic catheter).

|  | **unadjusted OR (95% CI)** | p-value | **adjusted OR (95% CI)** | p-value |
| --- | --- | --- | --- | --- |
| **Method of bladder emptying** |  | 0·76 |  |  |
| Transurethral catheter | 1·18 (0·41-3·39) |  | not applicable |  |
| Suprapubic catheter | reference |  | reference |  |
| **Sex** |  | 0.53 |  | 0·53 |
| Female | Reference |  | reference |  |
| Male | 0·72 (0·26-2·01) |  | 0·72 (0·26-2·01) |  |
| **CCI** |  | 0·17 |  | 0·15 |
| ≥ 3 | 3·59 (1·08-11·91) |  | 3·74 (1·12-12·52) |  |
| 2 | 2.·72 (0·66-11·17) |  | 2·92 (0·69-12·28) |  |
| 1 | 0 (0-999) |  | 0 (0-999) |  |
| 0 | reference |  | reference |  |
| **UTI frequency (in the past 12 months)** |  | 0·01 |  | 0·01 |
| ≥ 3 | 11·15 (2·63-47·22) |  | 11·63 (2·68-50·54) |  |
| 2 | 3·34 (0·7-15·93) |  | 3·54 (0·72-17·51) |  |
| 1 | 2·28 (0·55-9·49) |  | 2·34 (0·56-9·84) |  |
| 0 | reference |  | reference |  |
| **Age** | 0·98 (0·95-1·01) | 0·17 | 0·98 (0·95-1·01) | 0·15 |
| [CCI=Charlson comorbidity index](https://www.mdcalc.com/calc/3917/charlson-comorbidity-index-cci), CI=confidence interval, OR=odds ratio, UTI=urinary tract infection | | | | |

**Supplement Table 3**: Distribution of isolated bacteria in urine cultures in patients with an indwelling catheter.

| Bacterial distribution |  | Transurethral catheter | | Suprapubic catheter | |
| --- | --- | --- | --- | --- | --- |
|  | Total (N=509)  n (%) | UTI (N=16)  n (%) | ABU (N=117)  n (%) | UTI (N=21)  n (%) | ABU (N=355)  n (%) |
| *Enterococcus faecalis* | 113 (22%) | 5 (31%) | 24 (21%) | 6 (29%) | 78 (22%) |
| *Escherichia coli* | 64 (13%) | 1 (6%) | 15 (13%) | 2 (10%) | 46 (13%) |
| *Klebsiella* spp. | 47 (9%) | 2 (13%) | 16 (14%) | 2 (10%) | 27 (8%) |
| *Pseudomonas aeruginosa* | 35 (7%) | 1 (7%) | 7 (6%) | 2 (10%) | 25 (7%) |
| *Aerococcus urinae* | 29 (6%) | 0 | 4 (3%) | 0 | 25 (7%) |
| *Staphylococcus aureus* | 22 (4%) | 0 | 2 (2%) | 0 | 20 (6%) |
| *Proteus mirabilis* | 21 (4%) | 0 | 2 (2%) | 1 (5%) | 18 (5%) |
| *Streptococcus viridans* | 18 (4%) | 1 (7%) | 2 (2%) | 2 (10%) | 13 (4%) |
| *Staphylococcus epidermidis* | 12 (2%) | 1 (7%) | 9 (8%) | 0 | 2 (6%) |
| *Streptococcus anginosus* | 9 (2%) | 0 | 3 (3%) | 0 | 6 (2%) |
| others | 139 (27%) | 5 (31%) | 33 (28%) | 6 (29%) | 95 (27%) |

**Supplement Figure** 1: Urine culture characteristics for patient with an indwelling catheter


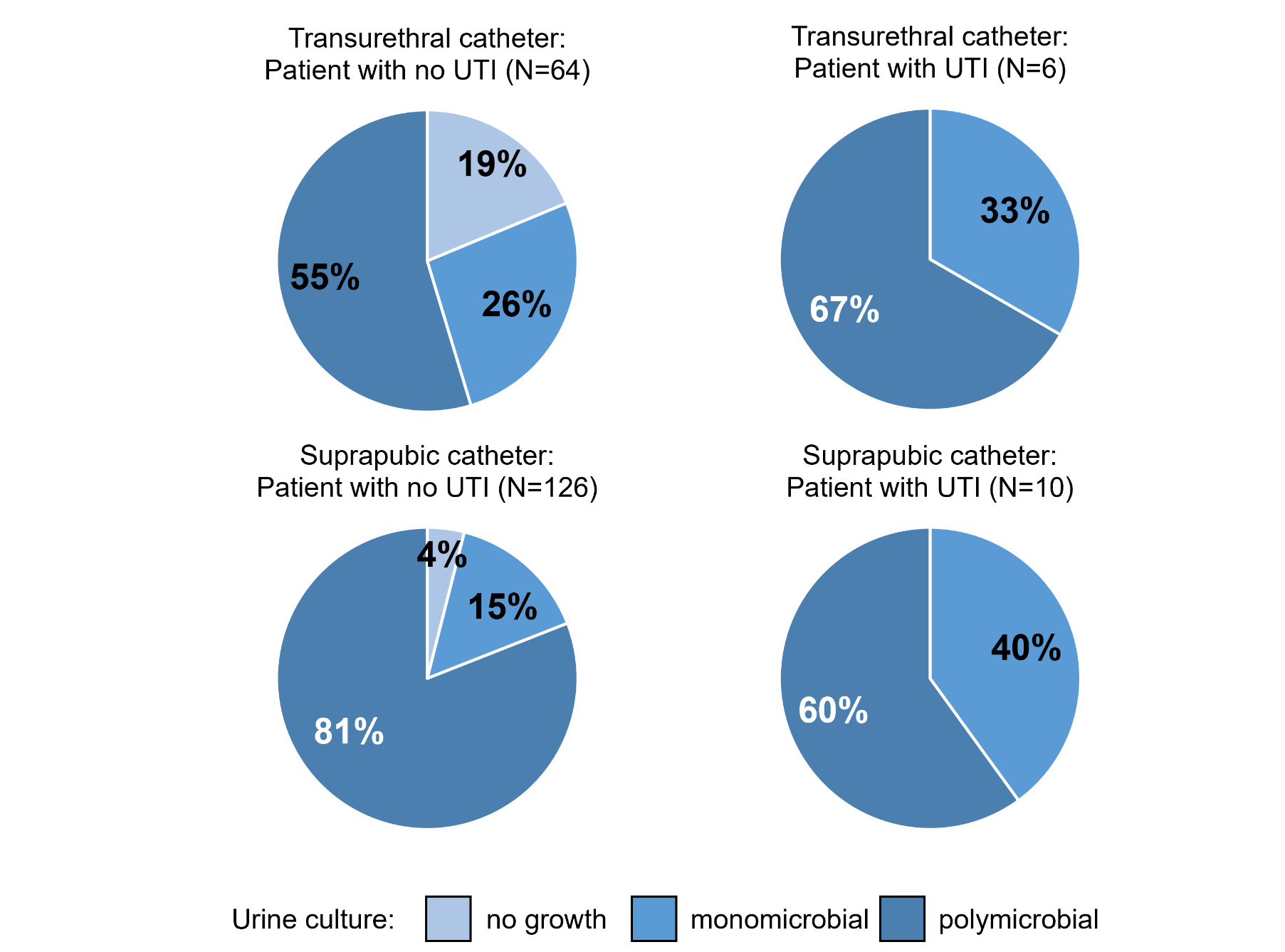
.

Legend: Urine culture results show percentages of patients without bacterial growth (no growth), detection of one species of bacteria (monomicrobial), or multiple bacterial species (polymicrobial) in patients with a transurethral and a suprapubic catheter.
